# Ventilatory function and cardiovascular disease risk factors: a cross-sectional study in young adults

**DOI:** 10.1186/1471-2466-14-206

**Published:** 2014-12-18

**Authors:** Vanessa Garcia-Larsen, Patricia Bustos, Hugo Amigo, James Potts, Roberto J Rona

**Affiliations:** Respiratory Epidemiology, Occupational Medicine, and Public Health Group, National Heart & Lung Institute Imperial College London, Emmanuel Kaye Building, Manresa Road, London, SW3 6LR UK; Department of Nutrition, Faculty of Medicine, University of Chile, Independencia, 1027 Santiago, Chile; Department of Psychological Medicine, 10 Cutcombe Road King’s College Hospital, London, SE5 9RJ UK

## Abstract

**Background:**

The association between impaired lung function and cardiovascular disease (CVD) risk factors has been shown in adults. However, there is little evidence of such an association in young adults, particularly from South America, where the burden of CVD and chronic obstructive pulmonary disease (COPD) is as high as that observed in more developed countries. We therefore investigated the relation between CVD risk factors including metabolic syndrome (MS), and lung function status in young adults from Chile.

**Methods:**

970 subjects from a sample of 998 adults born between 1974 and 1978 in Limache, Chile, were studied. A Spanish translation of the European Community Respiratory Health Survey (ECRHS) questionnaire was used. Forced expiratory volume in 1 second (FEV_1_) and forced vital capacity (FVC) were measured. Weight, height, waist circumference (WC), blood pressure, Homeostatic model assessment (HOMA-IR), triglycerides, high density lipoprotein (HDL), glycaemia, and metabolic syndrome (MS) were also assessed.

**Results:**

The prevalence of MS was 11.8%. A lower FEV_1_ and lower FVC were associated with having MS (β-coefficient -0.13; 95% Confidence Interval [CI] -0.21 to -0.05, and β-coefficient -0.18; 95% CI -0.27 to -0.09, respectively). Both spirometric measures were also negatively associated with having an elevated HOMA-IR (β-coefficient for FEV_1_ -0.08; 95% CI -0.13 to -0.03, and β-coefficient for FVC -0.11; 95% CI -0.17 to -0.05). In males only, a lower FEV_1_ and FVC were associated with having elevated triglycerides (β-coefficient highest vs. lowest tertile -0.13, 95% CI -0.24 to -0.03, and β-coefficient -0.13, 95% CI -0.25 to -0.01, respectively). In women, a higher FEV_1_ and FVC were statistically significantly related to having higher levels of HDL. Ventilatory function was unrelated to hypertension or WC in this population.

**Conclusion:**

In this population-based study of young adults, a poorer ventilatory function was associated with many CVD risk factors. Endeavours to understand better causality issues of such associations are warranted.

## Background

Poor lung function, usually defined as low forced expiratory volume in one second (FEV_1_) is a known risk factor for all-cause mortalities including respiratory and cardiovascular mortality [1].

Abdominal adiposity, insulin resistance, impaired glucose metabolism, hypertension and dyslipidaemia are known early markers of cardiovascular diseases (CVD) [2, 3]. Having three or more of these risk factors leads to metabolic syndrome (MS), a marker of systemic inflammation and a strong risk factor of CVD [3]. MS is a co-morbidity factor in as many as 50% of patients with COPD [4]. It has been proposed that Type 2 Diabetes Mellitus (T2DM) may act as an independent factor, negatively affecting pulmonary structure and function, increasing risk of pulmonary infections, disease exacerbations and worsened COPD outcomes [5]. Risk factors of CVD are frequently altered in childhood and adolescence [2]. However, the effect that this pattern might have in triggering the decline in ventilatory function has not yet been investigated in young individuals.

T2DM, MS and hypertension are usually silent and under-diagnosed conditions, especially in their early stages. Most of the evidence on the association between ventilatory function and risk factors of CVD comes from middle age and older adults, where there is increasing evidence of the detrimental effect that higher body fat and lower muscle mass have on the rate of lung function decline [6]. The aim of our study was to investigate the association between ventilatory function in young adults and risk factors of CVD, including MS.

## Methods

### Setting and sample

The study took place in Limache and Olmue, situated in the Central valley of Chile, 141 km from, Santiago, with a population of 50,000 inhabitants. Agriculture, and more recently ‘agro-tourism’ are the main economic activities in the area. This study is part of a non-concurrent longitudinal study aimed to assess risk factors in early childhood and in young adults for asthma and poor lung function [7].

A simple random sample of 1,232 subjects was obtained for our initial respiratory disease study based on statistical power estimates using the effects of birth weight differences on lung function as outcome for the respiratory study. For the cardiovascular study, a sample of 998 out of the 1,232 participants were studied based also on statistical power estimates and the resource implications of tracing the subjects already studied in the respiratory study which started a year earlier. Those who could not be included in the study because of death (3.2%, mostly related to maternal and infancy deaths in the early 70s), emigration (11.3%), serving a custodial sentence, disability or lactation (3.3%) and unwillingness to participate (7%) were randomly replaced using the same sampling frame [7].

### Lung function measurement

An adapted and translated version of the European Community Respiratory Health Survey (ECRHS) questionnaire was used to ascertain information on exposure to risk factors related to poor lung function [8]. FEV_1_ and forced vital capacity (FVC) were measured by trained nurses, using a Vitalograph device model 2120 Spirotrac IV. The nurses were unaware of the hypothesis being tested to minimise the risk of bias. These measurements were performed according to the recommendations from the American Thoracic Society (1987) because in Chile reference values have been published using these recommendations [9]. Any participant who was unable to produce two technically satisfactory manoeuvres after eight attempts was excluded from the study. Consequently, nine participants were removed from the study. The highest value for FEV_1_ and FVC produced in up to five satisfactory measures was used in the analyses. The FEV_1_/FVC ratio was also calculated. We used the National Health and Nutrition Examination Survey (NHANES) III prediction equations for white populations to estimate the lower limit of normal (LLN) of each spirometric measurement, which are calculated according to each individual’s age and sex [10]. We calculated the prevalence of airway obstruction in this population, estimating the proportion of individuals with a FEV_1_/FVC ratio below the LLN (bLLN) and the proportion of individuals with spirometric values bLLN for FEV_1_ and FVC too.

### Exposures

#### Lipids and HOMA-IR

Details of methods to calculate lipids and insulin resistance in this study population have been reported elsewhere [7]. Briefly, a 12-hour fasting blood sample was collected and serum was obtained for the measurement of insulin, and lipids. Insulin resistance status was estimated using the Homeostatic Model Assessment (HOMA-IR) method, which is calculated using concentrations of fasting glucose and insulin [11]. We used the cut-off value of 2.53, which has been defined for Chilean adults to indicate abnormal levels of insulin to maintain metabolic homeostasis [12]. For the measurement of glucose levels, a plasma sample with sodium fluoride was used. Insulin was determined by radioimmunoassay (Insulin kit, DPC, Los Angeles, USA) with a coefficient of variation of 7.9%. High density lipoprotein cholesterol (HDL) was determined by the Seigler & Wu method, with a variation coefficient of 4.6% [13]. Triglycerides were determined by an enzymatic method with clarifying factor (Human Diagnostic, Germany), with a coefficient of variation of 4.2%. All measurements were done by trained professionals within the same laboratory following the standards of the International Quality Control Programme of Bio-Rad Laboratories, Inc Hercules, CA. Lipid evaluation methods were carried out in line with the National Reference System for Cholesterol established by the Center for Disease Control and Prevention (CDC).

#### Metabolic syndrome (MS)

MS was diagnosed following the Adult Treatment Panel (ATP) III guidelines, according to which three or more of the following components must be present: abdominal obesity (waist circumference >102 cm in men and >88 cm in women), elevated blood pressure level (systolic blood pressure ≥ 130 mmHg or diastolic blood pressure ≥ 85 mmHg), elevated triglycerides (≥150 mg/dL), decreased HDL (<40 mg/dL in men and < 50 mg/dL in women), and elevated fasting glucose (≥ 110 mg/dL) [14].

#### Anthropometric measures

Waist circumference (WC), body weight, and height were measured by trained nurses. Body weight was recorded using a standard beam balance scale, with subjects barefoot and wearing light clothing [15]. Blood pressure was taken with a digital automatic sphygmomanometer, Omron 740, with a self-inflating cuff. The mean of the last two of three blood pressure measurements was used for this analysis. Waist circumference was measured on bare skin, midway between the lowest rib and the iliac crest, during mid-respiration on standing subjects. Anthropometric and blood pressure data were collected in local hospitals and health centres, using calibrated equipment and standardised methodology. Body mass index (BMI) was estimated as the ratio of weight to height squared (kg/m^2^).

#### Statistical analyses

We investigated the effect of risk factors of CVD on FEV_1_ and FVC as continuous outcomes of ventilatory function. The independent variables diastolic and systolic blood pressure, waist circumference, fasting glycaemia, triglycerides, and HDL were individually analysed in relation to each lung function outcome in the regression model. Exposure levels were analysed as tertiles with the exception of HOMA-IR and MS. These exposures are defined according to internationally agreed guidelines and were therefore analysed as binary variables normal/high, and absent/present, for HOMA-IR and MS, respectively. Given the high gender-related disparity in the proportion of individuals with prevalence of markers of CVD risk factors, the analyses are presented for the whole sample as well as by sex. Associations were considered of statistical significance if there was a p-value <0.05. As a measure to correct for possible multiple testing we considered statistically significant those associations with a p-value ≤ 0.01.

The following variables were included as potential confounders in the analyses of ventilatory function and risk factors of CVD: height, sex, age, smoking status, educational level, socio-economic status (SES), weight at birth, and BMI. Number of household belongings was used as a proxy for SES in this population (gas-fuelled water heater device, personal computer, fridge, washing machine, and microwave oven), which was appropriate to the reality of a semi-rural community in Chile [16].

We also carried out separate multi-variate analyses without BMI and smoking to explore whether there was an effect of these factors in the adjusted models. We observed that the associations were less strong after adjusting for BMI and smoking but remained statistically significant. Although BMI may be part of the chain of events which may explain the associations between CVD risk factors and ventilatory function, the rationale to adjust for BMI was to exclude this variable as a possible explanation for the associations in the analyses carried out. The results are presented including these confounders in the models. Analyses were carried out using STATA v12.0. The study was approved by the Ethics Committee of the Faculty of Medicine, University of Chile and from the St Thomas’s Hospital Ethics Committee in London.

## Results

The study sample comprised 970 young adults (mean age 25 years old) with complete lung function data and information on relevant exposures. Half of them were at least overweight, and almost two thirds (66.0%) of males and 49.2% of women reported being current smokers (Table 1).

**Table 1:** **Anthropometry, metabolic measurements and respiratory characteristics of participants**

Characteristics studied	Males (n = 429)	Females (n = 541)
***Age and Anthropometric measurements***		
Age (yr) Mean (SD)	25 (1.56)	24.7 (1.6)
Adult weight (kg) Median (IQR)	70.1 (63.1, 78.5)	62 (55, 70.6)
Adult height (cm) Mean (SD)	168 (6.1)	156.5 (5.7)
BMI (kg/m^2^) Median (IQR)	25 (11.7, 27.3)	25.1 (22.6, 28.8)
Weight at birth (g) Mean (SD)	3,201 (482.3)	3,178 (491.6)
***Measurements of lung function and lung obstruction***		
FEV_1_ (L)^*^ Median (IQR)	4.1 (3.8-4.5)	3.1 (2.8-3.4)
FVC (L)^*^ Median (IQR)	4.7 (4.3- 5.2)	3.5 (3.2-3.8)
FEV_1_ below LLN (n (%))	18 (4.2)	11 (2.0)
FVC below LLN (n (%))	32 (7.5)	14 (2.6)
***Potential confounders (n (%) unless indicated otherwise***		
Current smoker	283 (66.0)	266 (49.2)
Ex-smokers	37 (8.6)	70 (12.8)
12 years of full time education	330 (76.9)	394 (72.3)
Number of people with at least 4 of 6 household belongings (SES)	102 (23.8)	104 (18.9)
***Components of metabolic syndrome (n (%) unless indicated otherwise)***		
Metabolic syndrome (MS)^§^	53 (12.4)	61 (11.1)
HOMA-IR^#^	153 (35.8)	182 (33.2)
Abdominal obesity^&^	18 (4.2)	163 (29.7)
Fasting insulin (μU/mL) Mean (SD)	11.6 (5.8)	11.7 (5.8)
Hypertension^†^	41 (9.6)	13 (2.4)
High glycaemia^†^	6 (1.4)	2 (0.4)
High triglycerides^†^	96 (22.5)	75 (13.7)
Low HDL^†^	235 (54.9)	419 (76.3)

SD standard deviation.

SES Socio-Economic Status (No of household items used as an estimate of SES in the study population, namely having a washing machine, microwave, gas-fuelled water-heating device, refrigerator or a computer at home).

^*^Highest of five measurements.

^§^Metabolic syndrome (MS) – an individual was considered to have MS if three or more of the following components were present: 1) abdominal obesity (waist circumference > 102 cm in men and >88 cm in women); 2) elevated blood pressure (systolic blood pressure ≥ 130 mmHg or diastolic blood pressure ≥ 85 mmHg); 3) elevated triglycerides (≥ 150 mg/dL); 4) decreased high density lipoprotein cholesterol (HDL-C; < 40 mg/dL in men and < 50 mg/dL) and 5) elevated fasting glucose (≥ 110 mg/dL).

^#^HOMA-IR cut off point 2.53 or above considered high for Chilean adults; below 2.53 considered normal (reference value).

^&^Abdominal obesity defined as indicated in MS.

^†^Hypertension defined as indicated in MS (normal blood pressure as reference); high glycaemia, high triglycerides, and low HDL defined as indicated in MS.

As expected, men had higher lung volumes than women. 7.5% of men and 2.6% of women had an FVC bLLN. 29.7% of females and 4.2% of men had a waist circumference above recommendations for healthy status. Conversely, hypertension was more prevalent in men than in women (9.6% vs. 2.4%). Over a third of the population had elevated HOMA-IR (34.5%) and 11.8% had MS (Table 1).

Men had a statistically significantly lower FVC with increasing levels of triglycerides. Such an association was also observed in women but it was attenuated (p = 0.05). Similarly, men had a lower FVC with increasing tertiles of LDL levels, but this was not evident in women, who in turn, had a statistically significantly higher FEV_1_ and FVC with increasing levels of HDL (Table 2).

**Table 2:** **Association between markers of lipid metabolism and ventilatory function** ^*†^

Markers of lipid metabolism	Tertile	FEV _1_ (L) β-coefficient (95% CI)	FVC (L) β-coefficient (95% CI)
**Whole sample (n = 970)**			
Triglycerides	1	0	0
	2	0.03 (-0.03 to 0.09)	0.04 (-0.03 to 0.11)
	3	-0.03 (-0.09 to 0.04)	-0.03 (-0.10 to 0.05)
	*P value*	0.42	0.47
LDL	1	0	0
	2	0.03 (-0.08 to 0.04)	-0.01 (-0.09 to 0.06)
	3	-0.02 (-0.08 0.04)	-0.08 (-0.15 to -0.01)
	*P value*	0.56	**0.03**
HDL	1	0	0
	2	-0.04 (-0.01 to 0.12)	-0.03 (-0.11 to 0.04)
	3	0.10 (-0.01 to 0.12)	0.04 (-0.04 to 0.11)
	*P value*	0.10	0.34
**Men (n = 429)**			
Triglycerides	1	0	0
	2	-0.02 (-0.12 to 0.09)	-0.02 (-0.14 to 0.10
	3	-0.13 (-0.24 to -0.03)	-0.13 (-0.25 to -0.01)
	*P value*	**0.007**	**0.02**
LDL	1	0	0
	2	0.01 (-0.09 to 0.10)	-0.08 (-0.19 to 0.03)
	3	-0.05 (-0.15 to 0.05)	-0.16 (-0.27 to -0.04)
	*P value*	0.37	**0.006**
HDL	1	0	0
	2	-0.07 (-0.16 to 0.03)	-0.06 (-0.17 to 0.05)
	3	0.07 (-0.03 to 0.18)	0.03 (-0.10 to 0.14)
	*P value*	0.25	0.86
**Women (n = 541)**			
Triglycerides	1	0	0
	2	-0.02 (-0.08 to 0.04)	-0.02 (-0.09 to 0.05)
	3	-0.05 (-0.12 to 0.02)	-0.08 (-0.16 to -0.002)
	*P value*	0.16	0.05
LDL	1	0	0
	2	0.04 (-0.03 to 0.11)	0.04 (-0.03 to 0.12)
	3	0.01 (-0.05 to 0.08)	-0.01 (-0.08 to 0.07)
	*P value*	0.72	0.82
HDL	1	0	0
	2	0.02 (-0.04 to 0.09)	0.03 (-0.04 to 0.11)
	3	0.09 (0.02 to 0.15)	0.10 (0.03 to 0.17)
	*P value*	**0.01**	**0.007**

(*) Multiple linear regressions adjusted for age, gender, height, weight at birth, educational level, smoking, SES, and BMI; CI Confidence Interval.

^†^p-values in bold letters indicate statistical significance.

In the whole sample, a reduced FVC (110 ml) was associated with having an increased HOMA-IR. Stratified analyses by gender also showed evidence of a reduced FVC with an increased HOMA-IR in men and women (FVC reduced by 130 ml and 70 ml, respectively). Both FEV_1_ and FVC were negatively related to having MS in the whole sample, and in men. The association in women was only statistically significant between FVC and MS (Table 3). Subgroup analyses in never smokers showed no association between any of the exposures studied here and measures of lung function (data not shown).

**Table 3:** Association between glycaemia, HOMA-IR, metabolic syndrome (MS) and ventilatory function^*†^

Metabolic outcomes	Level compared	FEV _1_ (L) β-coefficient (95% CI)	FVC (L) β-coefficient (95% CI)
**Whole sample (n = 970)**			
Fasting glycaemia (tertile)	1	0	0
	2	0.01 (-0.06 to 0.07)	0.01 (-0.06 to 0.08)
	3	0.04 (-0.02 to 0.11)	0.03 (-0.04 to 0.10)
	*P value*	0.19	0.43
HOMA-IR	Normal	0	0
	High	-0.08 (-0.13 to -0.03)	-0.11 (-0.17 to -0.05)
	*P value*	**0.004**	**<0.0001**
Metabolic syndrome	Not present	0	0
	Present	-0.13 (-0.21 to -0.05)	-0.18 (-0.27 to -0.09)
	*P value*	**0.001**	**<0.001**
**Men (n = 429)**			
Fasting glycaemia (tertile)	1	0	0
	2	-0.01 (-0.12 to 0.09)	-0.001 (-0.13 to 0.12)
	3	-0.06 (-0.16 to 0.04)	-0.06 (-0.18 to 0.05)
	*P value*	0.24	0.25
HOMA-IR	Normal	0	0
	High	-0.12 (-0.21 to -0.03)	-0.13 (-0.23 to -0.03)
	*P value*	**0.007**	**0.01**
Metabolic syndrome	Not present	0	0
	Present	-0.18 (-0.32 to -0.05)	-0.20 (-0.35 to -0.04)
	*P value*	**0.009**	**0.01**
**Women (n = 521)**			
Fasting glycaemia (tertile)	1	0	0
	2	-0.002 (-0.06 to 0.06)	-0.03 (-0.07 to 0.06)
	3	0.01 (-0.06 to 0.08)	-0.03 (-0.11 to 0.04)
	*P value*	0.80	0.41
HOMA-IR	Normal	0	0
	High	-0.03 (-0.09 to 0.04)	-0.07 (-0.14 to -0.004)
	*P value*	0.43	**0.04**
Metabolic syndrome	Not present	0	0
	Present	-0.09 (-0.18 to 0.01)	-0.16 (-0.26 to -0.05)
	*P value*	0.08	**0.003**

(*) Multiple linear regressions adjusted for age, gender (where appropriate), height, weight at birth, educational level, smoking, SES, and BMI; Confidence Interval.

^†^p-values in bold letters indicate statistical significance.

We found no association between FEV_1_ or FVC and high blood pressure or increasing waist circumference. FEV_1_/FVC was not related to any of the exposures studied (data not shown).

## Discussion

We found a high prevalence of risk factors for MS and for cardio-vascular diseases (CVD) in this population of young adults. Having a lower lung function was associated with having HOMA-IR, dyslipidaemias and MS, but not with high blood pressure or waist circumference. Our study shows that the associations between abnormal lipid and insulin levels with lung function appear already in young adults, and that insulin resistance is present in individuals without declared T2DM.

An advantage of this study is the age group included, which allowed an evaluation of the association of early risk factors for CVD and ventilatory function. The assessment of respiratory outcomes in our study followed a rigorous international standardised protocol and quality control [8], which provided five high quality measurements of FEV_1_ and FVC. In addition, the response rate was high and participants were representative of the area where the research took place. To date, the epidemiological evidence available on the predisposing CVD risk factors on lower lung function in adults comes mostly from adults aged 40 or more [17–19].

Although the mortality for CVD has decreased in recent years in Latin America, it still remains in the first place of causes of death in this region of the world [20]. In spite of being the most important cause of the death, there is still limited knowledge on the distribution and prevalence of risk factors and CVD in Latin American countries. A recent study showed that the adult prevalence of hypertension is over 50% in this region, with Chile showing some of the highest prevalence rates for most CVD risk factors [21]. The epidemiological profile of general and specific mortality for COPD in South America is not very different to that of emerging middle income countries in other continents. It is known that lung function is a predictor of general mortality whilst COPD is currently the third cause of death worldwide [22]. However, the knowledge on lung function decline over time and its related risk factor in adults from developing countries, such as those of Latin America, is very limited.

In line with the high profile that CVD have in South America, we observed a high prevalence of risk factors for CVD, with nearly a third of the studied population having HOMA-IR, and a prevalence of MS of 11.8%. The current overall prevalence observed for these risk factors in Chile at country-level are the highest reported to date in South America [23].

There is epidemiological evidence showing an association between T2DM and MS with lower lung function in adults [24, 25]. Exposure to air pollutants, a known risk factor for COPD and reduced lung function, is unlikely to have affected the results of our study because levels of air pollution in Limache and Olmue are very low. Smoking is common in Chile and also in our study area. We have previously demonstrated that smoking in this sample only has a mild association with the FEV_1_/FVC ratio, but not with FVC_1_ and FVC [26]. Our results would indicate that in young adults, factors other than smoking, could explain a lower level of lung function. It is also possible that events occurring during foetal life [27], which have been proposed to affect the attainment of adult lung function, may explain these results.

In our study, women showed a statistically strong positive association between lung function and higher levels of HDL. Higher levels of HDL are known to contribute to the prevention of development of atherosclerosis. The benefits of higher concentrations of HDL expand to other metabolic-related functions, including preventing vascular inflammation and enhancing insulin sensitivity as well as promoting insulin secretion from the pancreas [28]. It might be possible that HDL could be further contributing to the maintenance of an improved lung function through their beneficial effects on other risk factors of MS.

The temporal relationship between MS or its main components, and lung function decline is difficult to disentangle [29]. MS encompasses several individual risk factors known to be involved in the activation of pro-inflammatory pathways in the lungs (e.g. dyslipidaemias, abdominal obesity), which might explain the negative associations found between MS, dyslipidaemias and outcomes of lung function. Our findings are in line to those reported from large population-based studies in adults from Europe. Leone *et al* found that all individual factors of MS were related to lung function impairment in adults with a mean age of 45 in France [19]. In a study sample of similar age range than that of the Limache Cohort, Li *et al* found that ‘pre-diabetes’ risk factors such as elevated fasting glucose and insulin resistance were associated with lower FVC [30]. Several studies have found a positive association between waist circumference with lung function, and other epidemiological studies have reported no association in relation to lung function or lung function decline in people with mild COPD [31]. Our findings rise the question of whether other lifestyle –related factors might explain the associations found.

Lack of physical activity and a sedentary lifestyle are related to a higher prevalence of dyslipidaemias, insulin resistance and obesity, all of which are known factors to increase the risk of CVD [32]. Given the young age group of this population, it would appear appropriate to propose health promotion strategies targeted to young adults in order to prevent or reduce MS risk factors. This might have an added value by contributing to slow down the decline in lung function. To date, smoking cessation is the only intervention shown to slow the decline of lung function. Our study provides evidence to justify the implementation of prospective studies to examine the possible impact of impaired markers of cardiovascular disease on lung function decline. Our results also justify intervention studies to investigate how modifying lifestyle behaviours that lead to prevent an increased insulin resistance, an unfavourable lipoprotein profile and MS, may reduce the losses of lung function over time.

A limitation of this study is its cross-sectional design, which prevents us from establishing a causal relationship between exposure to CVD risk factors and lung function decline. Full lung growth is usually reached after adolescence but it can continue developing in young adults, usually up to the age of 29 years old, or later if there is a history of pre-pubertal respiratory symptoms [33, 34]. The decline in lung function might start in the early 20s. In this study we are uncertain whether our results are due to CVD factors which reduce lung growth or induce an early loss of lung function. Longitudinal studies would allow us to examine how this relationship changes later in life and how poor lung function and MS interact and relate to CVD.

Efforts to understand the aetiological risk factors for CVD and COPD in Latin America have started in the last decade. The Limache Cohort study is one of the few cohorts to rigorously investigate early life risk factors for non-communicable diseases in adulthood. New large longitudinal studies are being set up in South America (including Chile) to investigate risk factors for CVD and poor lung function [35, 36].

Nutritional risk factors in the first year were unrelated to lung function in our studied population [37], and with the exception of an association with levels of lipoproteins measured in adulthood, nutritional status in the first year of life did not appear to be related to other CVD risk factors [38], These findings lend further support to the notion that exposure to environmental factors from childhood and adolescence might influence the trajectory of lung function.

## Conclusion

We found that poorer ventilatory function in young adults was associated with several markers of MS and with CVD risk factors. These findings provide a starting point to investigate mechanisms that could shed light to explain the association between lung function and CVD risk factors
